# Supplementary material for: Content-rich biological network constructed by mining PubMed abstracts
Source: BMC Bioinformatics. 2004 Oct 8;5:147. doi: 10.1186/1471-2105-5-147 (PMC528731; doi:10.1186/1471-2105-5-147)
Supplement: Additional File 2 — The original results of the above study (non-essential files are deleted to keep the file size under the limit set by BMC bioinformatics). [file 1471-2105-5-147-S2.bz2 › chilibotAdditionalFile2/dip30/341ID8076595E4256/html/TNFSF4_TNFRSF4.html]

 


 **TNFSF4** and **TNFRSF4** 
  
Found 114 abstracts in PubMed, retrieved 30.  
 

 What does Google say? 
 PDF only 
| .edu only 

---

**Interactive relationship** (e.g. stimulation, inhibition, etc)

**Inhibitory relationship**- Reverse transcriptase PCR analysis showed that expression of RANTES mRNA was induced after incubation with soluble OX40  [ **TNFRSF4** ]  and this induction was inhibited by anti gp34  [ **TNFSF4** ]  mAb.  Ref: 12161277 Immunol Lett, 2002
- Coculture with MMCE gp34  [ **TNFSF4** ]  inhibited the apoptosis of Kit225 OX40  [ **TNFRSF4** ] , but Kit225 del OX40  [ **TNFRSF4** ]  apoptosis was hardly affected.  Ref: 12416737 Int J Hematol, 2002
- Third, animals were treated with the fusion protein OX40  [ **TNFRSF4** ]  Ig which interferes with the OX40  [ **TNFRSF4** ]  OX40L  [ **TNFSF4** ]  interaction and thereby inhibits the intestinal manifestations of acute GvHD.  Ref: 11686535 Int J Colorectal Dis, 2001
- OX40  [ **TNFRSF4** ]  IgG fusion proteins act as decoys and inhibit T cell costimulation and extravasation induced by OX40  [ **TNFRSF4** ]  ligand  [ **TNFSF4** ]  expressing antigen presenting cells APC and vascular endothelial cells, respectively.  Ref: 12223520 J Leukoc Biol, 2002
**Neutral relationship**- Thus, OX40  [ **TNFRSF4** ]  OX40L  [ **TNFSF4** ]  interactions are pivotal to the efficiency of recall responses regulated by memory Th2 cells.  Ref: 12860930 J Exp MedJ Exp Med, 2002
- Interaction of OX40  [ **TNFRSF4** ]  CD134  [ **TNFRSF4** ]  and its ligand, OX40L  [ **TNFSF4** ] , has been implicated in T cell activation and cell migration.  Ref: 12794142 J Immunol, 2003
- In already sensitized animals, blocking OX40  [ **TNFRSF4** ]  OX40L  [ **TNFSF4** ]  interactions at the time of inhalation of aerosolized antigen suppressed memory effector accumulation in lung draining lymph nodes and lung, and prevented eosinophilia, airway hyperreactivity, mucus secretion, and Th2 cyto kine production.  Ref: 12860930 J Exp MedJ Exp Med, 2003
- Constitutive OX40  [ **TNFRSF4** ]  OX40  [ **TNFRSF4** ]  ligand  [ **TNFSF4** ]  interaction induces autoimmune like diseases.  Ref: 12370402 J Immunol, 2002
- The interaction between OX40  [ **TNFRSF4** ]  and OX40  [ **TNFRSF4** ]  ligand  [ **TNFSF4** ]  OX40L  [ **TNFSF4** ]  is suggested to provide T cells with an effective costimulatory signals during T cell APC interaction.  Ref: 12370402 J Immunol, 2002
- Blockade of OX40  [ **TNFRSF4** ]  OX40L  [ **TNFSF4** ]  interaction in the recipient RAG2 deficient mice completely prevented disease development.  Ref: 12370402 J Immunol, 2002
- Recently, several groups have reduced clinical signs of autoimmunity in animal models by blocking the OX40  [ **TNFRSF4** ]  OX40  [ **TNFRSF4** ]  ligand  [ **TNFSF4** ]  interaction or depleting OX40  [ **TNFRSF4** ]  T cells.  Ref: 11929124 Trends Immunol, 2002
- However, in vivo blockade of OX40  [ **TNFRSF4** ]  OX40L  [ **TNFSF4** ]  interactions reduced GVHD mortality induced by either signal transducer and activator of transcription 6 Stat 6 Th2 defective or Stat 4 Th1 defective major histocompatibility complex MHC disparate splenocytes, indicating that the GVHD ameliorating effects did not require Stat 4 or Stat 6 signaling.  Ref: 12521997 Blood, 2003
- The role of OX40  [ **TNFRSF4** ]  ligand  [ **TNFSF4** ]  interactions in the development of the Th2 response to the gastrointestinal nematode parasite Heligmosomoides polygyrus.  Ref: 12496423 J Immunol, 2003
- These studies suggest an important role for OX40  [ **TNFRSF4** ]  OX40L  [ **TNFSF4** ]  interactions in specifically promoting IL 4 production, as well as associated IgE elevations, in Th2 responses to H. polygyrus.  Ref: 12496423 J Immunol, 2003
- CD40KO mice primed with C26 GM OX40L  [ **TNFSF4** ]  cells failed to mount a CTL response, and T cells infiltrating the C26 GM OX40L  [ **TNFSF4** ]  tumor were OX40  [ **TNFRSF4** ]  negative, suggesting an impairment in APC T cell cross talk in CD40KO mice.  Ref: 12496388 J Immunol, 2003
- Interaction of OX40  [ **TNFRSF4** ]  CD134  [ **TNFRSF4** ]  on T cells with its ligand OX40L  [ **TNFSF4** ]  on antigen presenting cells has been implicated in pathogenic T cell activation.  Ref: 12631559 Am J Physiol Gastrointest Liver Physiol, 2003
- To examine the in vivo effect of constitutive OX40  [ **TNFRSF4** ]  OX40L  [ **TNFSF4** ]  interaction during immune regulation, we report the establishment of OX40L  [ **TNFSF4** ]  transgenic OX40L  [ **TNFSF4** ]  Tg mice that constitutively express OX40L  [ **TNFSF4** ]  on T cells.  Ref: 12370402 J Immunol, 2002
- In addition, OX40  [ **TNFRSF4** ]  IgG1 protein induces ADCC and complement lysis in OX40  [ **TNFRSF4** ]  ligand  [ **TNFSF4** ]  expressing cells.  Ref: 12223520 J Leukoc Biol, 2002
- BACKGROUND The CD134  [ **TNFRSF4** ]  CD134L  [ **TNFSF4** ]  interaction provides a strong costimulatory signal that is CD28 independent.  Ref: 12134113 Transplantation, 2002
- These findings indicate that 1 resident CD69 OX40  [ **TNFRSF4** ]  IELs constitute a population of partially activated T cells poised for rapid delivery of effector activity, 2 OX40  [ **TNFRSF4** ]  and OX40L  [ **TNFSF4** ]  expression defines IELs that have undergone recent immune activation, 3 OX40  [ **TNFRSF4** ]  IELs are significantly more efficient CTL than are OX40  [ **TNFRSF4** ]  IELs, and 4 the local OX40  [ **TNFRSF4** ]  OX40L  [ **TNFSF4** ]  system plays a critical role in regulating the magnitude of cytokine responses in the gut epithelium.  Ref: 11739485 J Immunol, 2001
- These results suggest that the OX40  [ **TNFRSF4** ]  OX40L  [ **TNFSF4** ]  system is crucial for induction of the allogeneic T cell response and the OX40  [ **TNFRSF4** ]  OX40L  [ **TNFSF4** ]  system is subsequent to and dependent on CD28 signalling, but is crucial for the end outcome of the human alloreactive T cell response.  Ref: 12757617 Immunology, 2003
- CONCLUSION These results present the first evidence that the CD134  [ **TNFRSF4** ]  CD134L  [ **TNFSF4** ]  interaction is associated with acute allograft rejection.  Ref: 12134113 Transplantation, 2002
- These results clearly indicate that gp34  [ **TNFSF4** ]  signaling induces expression of RANTES at both mRNA and protein levels in HUVECs and suggest a possible link between the OX40  [ **TNFRSF4** ]  gp34  [ **TNFSF4** ]  system and RANTES during the process of T cell adhesion to endothelial cells and subsequent extravasation.  Ref: 12161277 Immunol Lett, 2002
- HUVECs expressing high levels of gp34  [ **TNFSF4** ]  were stimulated with recombinant soluble OX40  [ **TNFRSF4** ]  or mock control and subjected to analysis using cDNA expression arrays.  Ref: 12161277 Immunol Lett, 2002

**Non-interactive relationship** (e.g. studied together, co-existance, homology, etc.)

- Immunohistochemistry and flow cytometry were used to analyze the expression and localization of CD134  [ **TNFRSF4** ]  CD134L  [ **TNFSF4** ] .  Ref: 12134113 Transplantation, 2002
- However, OX40  [ **TNFRSF4** ]  and OX40L  [ **TNFSF4** ]  expression were rapidly up regulated on CD8 IELs following CD3 stimulation, indicating that both markers on IELs reflect activation dependent events.  Ref: 11739485 J Immunol, 2001
- In this study, we show that engagement of OX40  [ **TNFRSF4** ]  by an OX40L  [ **TNFSF4** ]  Ig fusion protein drives IFN gamma production by CD4 T cells and reduces eosinophilia and C. neoformans burden in the lung.  Ref: 12794142 J Immunol, 2003
- Combined CD134  [ **TNFRSF4** ]  CD134L  [ **TNFSF4** ]  blockade may be an effective treatment to both prevent acute allograft rejection and promote the induction of cells with regulatory capacity.  Ref: 12134113 Transplantation, 2002
- To investigate the roles of the co stimulatory molecules CD30 CD30L, CD27 CD27L, OX40  [ **TNFRSF4** ]  OX40L  [ **TNFSF4** ] , and 4 1BB 4 1BBL, which belong to the tumor necrosis factor  [ **TNFSF4** ]  TNF receptor ligand superfamily, in the development of acute viral myocarditis, the expression of these molecules was first analysed in the hearts of mice with acute myocarditis induced by Coxsackievirus B3 CVB3 in vivo.  Ref: 11745696 J Pathol, 2001
- Consequences of OX40  [ **TNFRSF4** ]  OX40  [ **TNFRSF4** ]  ligand  [ **TNFSF4** ]  interactions in langerhans cell function enhanced contact hypersensitivity responses in OX40L  [ **TNFSF4** ]  transgenic mice.  Ref: 12555678 Eur J Immunol, 2002
- Although freshly isolated CD8 IELs exhibited properties of activated T cells CD69 expression and ex vivo cytotoxicity, virtually all CD8 IELs from normal mice were devoid of other activation associated properties, including a lack of expression of OX40  [ **TNFRSF4** ]  and the ligand for OX40  [ **TNFRSF4** ]  OX40L  [ **TNFSF4** ]  and an absence of intracellular IFN gamma staining.  Ref: 11739485 J Immunol, 2001
- its ligand, OX40  [ **TNFRSF4** ]  ligand  [ **TNFSF4** ]  OX40L  [ **TNFSF4** ]  is expressed on dendritic cells, B cells, and activated endothelial cells.  Ref: 12521997 Blood, 2003
- In these studies, we examined the effects of OX40  [ **TNFRSF4** ]  ligand  [ **TNFSF4** ]  OX40L  [ **TNFSF4** ]  deficiency on the development of Th2 cells during the Th2 immune response to the intestinal nematode parasite Heligmosomoides polygyrus.  Ref: 12496423 J Immunol, 2003
- Our data suggest that CD134  [ **TNFRSF4** ]  CD134L  [ **TNFSF4** ]  is a critical pathway in alloimmune responses, especially recall primed responses, and is synergistic with CD28 B7 in mediating T cell effector responses during allograft rejection.  Ref: 12626546 J Immunol, 2003
- OX40  [ **TNFRSF4** ]  ligand  [ **TNFSF4** ]  transduced tumor cell vaccine synergizes with GM CSF and requires CD40 Apc signaling to boost the host T cell antitumor response.  Ref: 12496388 J Immunol, 2003
- ... blockade of OX40  [ **TNFRSF4** ]  OX40L  [ **TNFSF4** ]  by anti OX40L  [ **TNFSF4** ]  mAb markedly increased IL 10 production.  Ref: 11739485 J Immunol, 2001
- To determine how OX40  [ **TNFRSF4** ]  OX40L  [ **TNFSF4** ]  interaction affects graft versus host disease GVHD, we used antagonistic anti OX40L  [ **TNFSF4** ]  monoclonal antibody mAb or OX40  [ **TNFRSF4** ]  donor or OX40L  [ **TNFSF4** ]  recipient mice.  Ref: 12521997 Blood, 2003
- METHODS CD134  [ **TNFRSF4** ]  CD134L  [ **TNFSF4** ]  costimulation was studied in a rat model of small bowel transplantation.  Ref: 12134113 Transplantation, 2002
- The costimulatory molecule OX40  [ **TNFRSF4** ]  ligand  [ **TNFSF4** ]  OX40L  [ **TNFSF4** ] , a member of the tumor necrosis factor  [ **TNFSF4** ]  superfamily, has been shown to be important in T cell priming and cytokine production.  Ref: 12355440 Eur J Immunol, 2002
- In the present study, we report the expression of both OX40L  [ **TNFSF4** ]  and OX40  [ **TNFRSF4** ]  on differentiated LC derived from draining lymph nodes in the FITC sensitized mice.  Ref: 12555678 Eur J Immunol, 2002
- To determine whether a cellular vaccine that interacts directly with both APC and T cells in vivo might be superior, we generated C26 carcinoma cells transduced with the T cell costimulatory molecule OX40  [ **TNFRSF4** ]  ligand  [ **TNFSF4** ]  OX40L  [ **TNFSF4** ]  either alone C26 OX40L  [ **TNFSF4** ]  or together with GM CSF C26 GM OX40L  [ **TNFSF4** ] , which is known to activate APC.  Ref: 12496388 J Immunol, 2003
- We first demonstrated that anti OX40  [ **TNFRSF4** ]  ligand  [ **TNFSF4** ]  anti OX40L  [ **TNFSF4** ]  monoclonal antibody mAb could markedly suppress the mixed leucocyte reaction MLR of peripheral blood mononuclear cells PBMC.  Ref: 12757617 Immunology, 2003
- OX40  [ **TNFRSF4** ]  CD134  [ **TNFRSF4** ] , a membrane bound member of the tumor necrosis factor  [ **TNFSF4** ]  receptor superfamily, is expressed primarily on activated CD4 T cells.  Ref: 11929124 Trends Immunol, 2002
- The present results orchestrated in this study indicate that OX40  [ **TNFRSF4** ]  OX40L  [ **TNFSF4** ]  interaction may be a vital link in our understanding of T cell mediated organ specific autoimmunity.  Ref: 12370402 J Immunol, 2002
- Unlike dendritic cells, the CD4 CD3 cells have little CD80 or CD86 expression but do express high levels of the TNF ligands, OX40  [ **TNFRSF4** ]  ligand  [ **TNFSF4** ]  and CD30 ligand.  Ref: 12753741 Immunity, 2003
- We recently demonstrated a critical contribution of OX40  [ **TNFRSF4** ]  ligand  [ **TNFSF4** ]  OX40L  [ **TNFSF4** ]  to the development of Th2 mediated experimental leishmaniasis.  Ref: 12672051 Eur J Immunol, 2003
- These results indicate that serial measurement of OX40  [ **TNFRSF4** ]  T cells is useful for predicting the onset as well as the therapeutic response of cGVHD and raise a possibility that the OX40  [ **TNFRSF4** ]  gp34  [ **TNFSF4** ]  system is involved in the pathogenesis of cGVHD.  Ref: 11698307 Blood, 2001
- To further define the contribution of the OX40  [ **TNFRSF4** ]  OX40L  [ **TNFSF4** ]  system to the MLR, we set up a co culture system of CD4 T cells and allogeneic monocyte derived dendritic cells DCs.  Ref: 12757617 Immunology, 2003
- Protein extract derived from the helminth Schistosoma mansoni induced the development of DC2s that promote the development of Th2 cells via the enhanced expression of OX40  [ **TNFRSF4** ]  ligand  [ **TNFSF4** ] .  Ref: 11823500 J Immunol, 2002
- RESULTS CD134  [ **TNFRSF4** ]  and CD134L  [ **TNFSF4** ]  molecules were strongly expressed in acutely rejected small bowel allografts.  Ref: 12134113 Transplantation, 2002
- Critical role for OX40  [ **TNFRSF4** ]  ligand  [ **TNFSF4** ]  in the development of pathogenic Th2 cells in a murine model of asthma.  Ref: 12672051 Eur J Immunol, 2003
- GVHD inhibition by blockade of the OX40  [ **TNFRSF4** ]  OX40L  [ **TNFSF4** ]  pathway did not require CD28 signaling.  Ref: 12521997 Blood, 2003
- In all these patients, the coculture of ATL cells with MMCE OX40  [ **TNFRSF4** ]  ligand  [ **TNFSF4** ]  gp34  [ **TNFSF4** ] , a stable human gp34  [ **TNFSF4** ]  transfectant of a mouse epithelial cell line, resulted in a decrease in the percentage of apoptotic cells after treatment with anti Fas monoclonal antibody, compared to coculture with MMCE mock controls.  Ref: 12416737 Int J Hematol, 2002
- This study was performed to explore the involvement of OX40  [ **TNFRSF4** ]  OX40L  [ **TNFSF4** ]  in the development of T cell mediated chronic colitis.  Ref: 12631559 Am J Physiol Gastrointest Liver Physiol, 2003
- OX40  [ **TNFRSF4** ]  ligand  [ **TNFSF4** ]  OX40L  [ **TNFSF4** ] , a member of TNF superfamily, is expressed on several APC such as splenic dendritic cells DC and activated B cells.  Ref: 12555678 Eur J Immunol, 2002
- However, B cell costimulation via the OX40L  [ **TNFSF4** ]  OX40  [ **TNFRSF4** ]  pathway that has been implicated in CD4 cell expansion, survival, and Th2 development was required.  Ref: 12668647 J Exp Med, 2003
- Our present results suggested a pivotal role of OX40  [ **TNFRSF4** ]  OX40L  [ **TNFSF4** ]  in the pathogenesis of T cell mediated chronic colitis.  Ref: 12631559 Am J Physiol Gastrointest Liver Physiol, 2003
- Signaling of gp34  [ **TNFSF4** ]  OX40  [ **TNFRSF4** ]  ligand  [ **TNFSF4** ]  induces vascular endothelial cells to produce a CC chemokine RANTES CCL5.  Ref: 12161277 Immunol Lett, 2002
- The CD134  [ **TNFRSF4** ]  CD134  [ **TNFRSF4** ]  ligand  [ **TNFSF4** ]  CD134L  [ **TNFSF4** ]  costimulatory pathway has been shown to be critical for both T and B cell activation.  Ref: 12626546 J Immunol, 2003
- Furthermore, in OX40L  [ **TNFSF4** ]  Tg mice administrated with superantigen, this constitutive OX40  [ **TNFRSF4** ]  OX40L  [ **TNFSF4** ]  interaction on CD4 T cells completely prevented normal in vivo clonal T cell deletion.  Ref: 12370402 J Immunol, 2002
- We reported previously that OX40  [ **TNFRSF4** ] , a member of the tumor necrosis factor  [ **TNFSF4** ]  receptor family, is expressed constitutively on fresh leukemia lymphoma cells isolated from patients with adult T cell leukemia ATL.  Ref: 12416737 Int J Hematol, 2002
- Likewise, toxin from the extracellular bacterium Vibrio cholerae induced development of DC2s as well via an OX40  [ **TNFRSF4** ]  ligand  [ **TNFSF4** ]  independent, still unknown mechanism.  Ref: 11823500 J Immunol, 2002
- This study looks at whether the expression of OX40  [ **TNFRSF4** ] , a member of the tumor necrosis factor  [ **TNFSF4** ]  receptor family, is related to the development of cGVHD in patients who underwent allogeneic hematopoietic stem cell transplantation.  Ref: 11698307 Blood, 2001
- Unlike IELs, activated lymph node T cells did not express OX40L  [ **TNFSF4** ] , thus indicating that OX40  [ **TNFRSF4** ]  OX40L  [ **TNFSF4** ]  communication in the intestinal epithelium is part of a novel CD8 network.  Ref: 11739485 J Immunol, 2001
- We investigated the effect of CD134  [ **TNFRSF4** ]  CD134L  [ **TNFSF4** ]  pathway blockade on allograft rejection in fully MHC mismatched rat cardiac and skin transplantation models.  Ref: 12626546 J Immunol, 2003
- Crucially, the up regulation of OX40L  [ **TNFSF4** ]  on antigen presenting cells and CXCR 5 on T cells are CD40 dependent events and we show that T cells do not migrate to follicles in immunized OX40  [ **TNFRSF4** ]  deficient mice.  Ref: 12538659 J Exp Med, 2003
- These data provide insights as to the mechanisms responsible for OX40  [ **TNFRSF4** ]  OX40L  [ **TNFSF4** ]  regulation of GVHD.  Ref: 12521997 Blood, 2003
- We previously showed that gp34  [ **TNFSF4** ]  OX40  [ **TNFRSF4** ]  ligand  [ **TNFSF4** ]  expressed on vascular endothelial cells is not only involved in adhesion between activated T cells and endothelial cells but also by itself able to transmit intracellular signals leading to expression of c fos and c jun mRNA upon OX40  [ **TNFRSF4** ]  binding.  Ref: 12161277 Immunol Lett, 2002
- Association of the CD134  [ **TNFRSF4** ]  CD134L  [ **TNFSF4** ]  costimulatory pathway with acute rejection of small bowel allograft1.  Ref: 12134113 Transplantation, 2002
- The CD134  [ **TNFRSF4** ]  CD134L  [ **TNFSF4** ]  pathway has been studied in inflammatory, autoimmune diseases, and graft versus host disease, but no information exists on the involvement of CD134  [ **TNFRSF4** ]  CD134L  [ **TNFSF4** ]  interactions in solid organ transplantation.  Ref: 12134113 Transplantation, 2002
- The Role of the CD134  [ **TNFRSF4** ]  CD134  [ **TNFRSF4** ]  Ligand  [ **TNFSF4** ]  Costimulatory Pathway in Alloimmune Responses In Vivo.  Ref: 12626546 J Immunol, 2003
